# Status of meat alternatives and their potential role in the future meat market — A review

**DOI:** 10.5713/ajas.20.0419

**Published:** 2020-07-28

**Authors:** Hyun Jung Lee, Hae In Yong, Minsu Kim, Yun-Sang Choi, Cheorun Jo

**Affiliations:** 1 Department of Agricultural Biotechnology, Center for Food and Bioconvergence, and Research Institute of Agriculture and Life Science, Seoul National University, Seoul 08826, Korea; 2 Research Group of Food Processing, Korea Food, Research Institute, Wanju 55365, Korea; 3 Institute of Green Bio Science and Technology, Seoul, National University, Pyeongchang 25354, Korea

**Keywords:** Meat Alternatives, Plant-based Meat Analogues, Edible Insects, Cultured Meat, Protein Sources

## Abstract

Plant-based meat analogues, edible insects, and cultured meat are promising major meat alternatives that can be used as protein sources in the future. It is also believed that the importance of meat alternatives will continue to increase because of concerns on limited sustainability of the traditional meat production system. The meat alternatives are expected to have different roles based on their different benefits and limitations. Plant-based meat analogues and edible insects can replace traditional meat as a good protein source from the perspective of nutritional value. Furthermore, plant-based meat can be made available to a wide range of consumers (e.g., as vegetarian or halal food products). However, despite ongoing technical developments, their palatability, including appearance, flavor, and texture, is still different from the consumers’ standard established from livestock-based traditional meat. Meanwhile, cultured meat is the only method to produce actual animal muscle-based meat; therefore, the final product is more meat-like compared to other meat analogues. However, technical difficulties, especially in mass production and cost, remain before it can be commercialized. Nevertheless, these meat alternatives can be a part of our future protein sources while maintaining a complementary relationship with traditional meat.

## INTRODUCTION

Meat can be defined as “the flesh of an animal destined for our consumption as food” and includes edible parts of animal carcass, such as lean meat, fat, intestines, etc. [1,2]. Historically, as a food resource, meat has contributed to human evolution and development [3]. Meat is composed of essential nutrients, especially proteins, which are necessary for various physiological functions in the human body [4]. It provides approximately 15% of the proteins consumed in our diet and contains all the essential amino acids as well as various fatty acids and micronutrients (e.g., vitamin B complex, Fe, Zn, and Se) [5,6]. Moreover, meat protein has high digestibility with a corrected amino acid score reaching 0.92 [3]. In addition, it is flavorful and known to have important social and cultural meanings in human society [7–9]. Therefore, without doubt, meat is not only an important food for humans but is also an essential part of our lives.

Currently, the world population is growing fast and will reach 9 billion by 2050 [10]. It is estimated that we will need at least doubled amounts of meat compared to those we are producing now. This rapid increase in the global demand for meat is attributed not only to population growth, but also to economic development of developing countries [11,12]. Taking these factors into consideration, we have to shortly find a way to increase the production of meat. Earlier, industrialization of livestock farming fulfilled the increasing demand for meat and its products [10]. However, it is no longer possible to increase meat production for future demands because of the limited land and water resources for sustainability of livestock farming, rapid increase in animal welfare issues, and undesirable impact on the environment and climate changes [1]. Based on the gap between future demand and the present capability to supply meat, there is an increasing need for producing meat alternatives as protein sources. Furthermore, expansion of halal and kosher markets will also require the development of meat alternatives instead of livestock-based traditional meat, as the number of people consuming such foods might exceed 30% of the world population by 2025 [13].

Consequently, several efforts have been made to increase the production of conventional meat and/or different meat alternatives (Table 1) [10]. Among them, plant-based meat analogues, edible insects, and cultured meat are garnering the interest of most consumers, although cultured meat is still under development for commercialization. Therefore, in this review, the major meat alternatives (e.g., plant-based meat analogues, edible insects, and cultured meat) are introduced as promising protein sources that can be utilized in the near future for supporting and complementing the limited sustainability of the traditional meat production system.

## PLANT-BASED MEAT ANALOGUES

### Definition and present features

Plant-based meat analogues can be manufactured using protein extracted from plants [10]. Wheat, soybean, legumes, oil seeds, and fungi are known to be the main sources of plant-based meat analogues (Table 2) [13]. In fact, plant protein is one of the oldest food sources in our history. Tofu was first consumed in 965 CE, and several products, including wheat gluten, yuba, and tempeh, have been used for decades in different countries and regions [14,15]. Moreover, plant-based products have been suggested as a meat substitute since 1888. However, most of them had very different characteristic features compared to traditional meat, especially with respect to flavor and texture. Therefore, these products did not succeed in the market until the 1900s: the consumption of plant-based meat analogues was only limited to its economic benefits and social demands related to health, religion, and ethical reasons; however, its consumption did not have a pleasurable effect as far as flavor and texture were concerned [13].

In recent times, the market for plant-based meat analogues is expanding with increasing social demands, and constant efforts are being taken to improve their sensory qualities [14, 15]. Introduction of texturized vegetable protein (TVP) produced using various ingredients led to the development of plant-based meat analogues; currently, it occupies the biggest market among the different meat alternatives, and it is believed that the market will increase to over $21.23 billion US dollars by 2025 [14,15].

### Benefits as meat alternatives

The major reason for meat consumption is to obtain nutrition [13]. Thus, it is very important to manufacture plant-based meat analogues to meet the nutrient specifications of traditional meat [16]. In general, plant protein is limited in nutritional value because of the lack of several essential amino acids such as lysine, methionine, and/or cysteine, and has low bioavailability [17].

Based on their nutritional values and functions, wheat gluten and soybean proteins are the most-used sources among different plant proteins to prepare plant-based meat analogues [13]. Wheat, containing 8% to 17.5% proteins, is one of the most important crops. Gluten (subdivided into gliadin and glutenin) from wheat can be produced during the wet processing of flour and is approved as “Generally Recognized as Safe” (GRAS) grade. When it is heated above 85°C, gluten can be coagulated, resulting in gel formation without loss of its structural order. Moreover, as gluten can form a cohesive blend between protein and the other ingredients, it can be utilized as a plant protein to produce meat analogues. Meanwhile, soybean protein is derived from leguminous plants, as are clover, peas, and alfalfa. It is recently attracting the interest of consumers as a good protein source with economic benefits. Malav et al [18] reported that soybeans have 35% to 40% of high-quality proteins, 15% to 20% of fats, 30% carbohydrates, as well as Fe, Ca, Zn, and vitamin B groups. Liu et al [19] suggested that soybean protein can be used as an alternative to meat products because of its excellent capacity for rehydration, oil absorption, emulsification, and water absorption.

**Figure 1:**
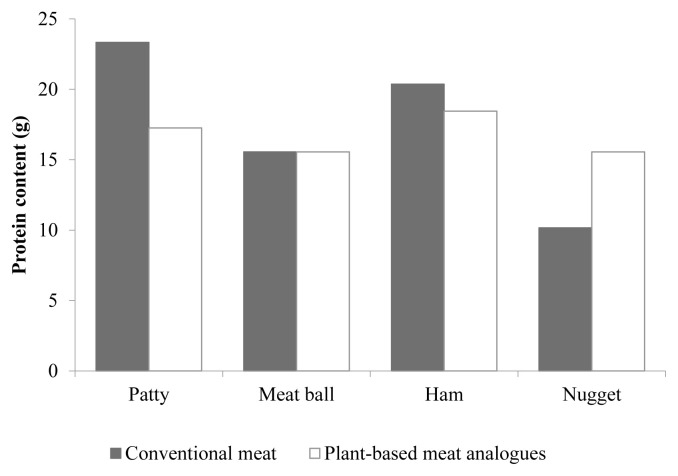
Protein content (g) in four different types of traditional meat and plant-based meat analogues in market. Modified from Bohrer [14].

In the current market, several products are successful as plant-based meat analogues and seem to provide sufficient amount of proteins to our diet as meat alternatives. Bohrer [14] investigated the nutritional contents in four major types (beef burger products, beef meatballs, pork ham, and chicken nuggets) of traditional meat and plant-based meat analogues in market. They found that each beef patty in a burger contains 23.33 g of protein, whereas a meat analogue patty has approximately 19.46 g of protein (Figure 1). However, plant-based meat analogues have less cholesterol and more dietary fiber, which can be appealing to consumers. The other types of products (beef meatballs, pork ham, and chicken nuggets) also showed similar overall results (See Bohrer [14] for more detailed information). Therefore, as far as nutritional aspects are concerned, especially the protein contents, plant-based meat analogues are likely to be good substitutes to traditional meat. The products will be beneficial to consumers who cannot eat traditional meat and meat products, mostly owing to their religious and ethical beliefs. In particular, when considering the massive market expansion for halal and kosher food products as well as the increasing interest in animal welfare, among the various meat alternatives, protein sources devoid of animal protein will be in high demand as plant-based meat analogues in the future.

### Research trends and challenges

Despite the good nutritional value and continuous development of plant-based meat analogues, their palatability remains a critical obstacle for consumer acceptability. For improving the texture and flavor of plant-based meat analogues, different ingredients are added during the manufacturing process (Table 3). Regarding texture, different techniques such as spinning, thermoplastic extrusion, and steam texturization have also been applied for the structural organization of plant protein, as plants are mainly composed of amorphous tissue [20,21]. Among these, extrusion is the most frequently used technique, as it is an economical method and can manufacture different shapes and sizes of meat analogues. The process is based on a screw system within a barrel [22] by means of which plant proteins are compressed, heated to be restructured into a striated, layered, and cross-linked mass, ultimately leading to the production of TVP [13,23]. Previous research suggested that utilizing wheat gluten and soybean protein as TVP ingredients could impart an appearance, texture, taste, and nutritional value similar to that of traditional meat [24]. In addition, proteins produced from starch by-products using fungi (a.k.a. mycoprotein) have structures and diameters similar to those of muscle fibers of meat with almost a similar texture [13,21].

The flavor of traditional meat is mainly derived from flavor-related compounds such as free amino acids, free fatty acids, nucleotides, and reducing sugars. Besides, vitamin B1 and myoglobin also affect the flavor of meat [25]. Therefore, when plant-based meat analogues are produced, flavor enhancers are added (Table 3). According to Kyriakopoulou et al [26], when volatile compounds in traditional meat are isolated after a combination of various thermal processes, a flavor concentrate of meat is obtained. Subsequently, different techniques have been investigated and developed to incorporate such flavor concentrates into plant-based meat analogues to achieve a meat flavor. Addition of fat/oil (e.g., canola oil, coconut oil, and sunflower oil) can also affect the formation of flavor in plant-based meat analogues as well as their texture and mouthfeel [13,14].

Another challenge for plant-based meat analogues is the appearance, especially color. The color of meat and meat analogues is an important attribute at the point of purchase in the market [27]. To represent the color of red meat, some meat analogue products contain beet juice extract or tomato paste [14]. However, meat color does not always appear red, and it changes depending on the chemical state of myoglobin, which is primarily responsible for the meat color. Despite fresh meat possessing a bright red color due to high oxymyoglobin content, the meat color changes to brown, and metmyoglobin content increases when meat is cooked [28]. Some researchers have proposed that meat analogues should have color attributes similar to those of traditional raw or cooked meat [26]. Thus, the meat industry produces and uses leghemoglobin, which has a similar chemical state and structure as myoglobin. A representative product containing leghemoglobin is the Impossible Burger (Impossible Foods Inc., Redwood City, CA, USA). When leghemoglobin is added to a meat analogue product, it imparts cooked-color characteristics similar to those of traditional meat [14,29]. Myoglobin also affects meat flavor. Thus, Fraser et al [30] reported that the use of leghemoglobin, which is similar to myoglobin, provided a distinct meat flavor to meat analogues. In addition, leghemoglobin was shown to be free of toxicity as examined by *in vitro* chromosomal aberration tests and *in vivo* systemic toxicity test [30].

As plant protein and food-grade ingredients are mainly used during manufacture of plant-based meat analogues, their safety is approved, and production cost is feasible [31]. However, several anti-nutrients (e.g., protease inhibitors, α-amylase inhibitors, lectin, polyphenols, and phytic acid) are present in plant-based meat analogues. Although these compounds are known for their positive effects, such as anticarcinogenic, anti-obesity, lymphocyte stimulation, antioxidant effects, and others, their negative effects have also been reported [13]. For example, polyphenols can decrease the activities of digestive enzymes as well as bioavailability of proteins and amino acids. Phytic acid can induce mineral depletion and micronutrient deficiency as it reduces the bioavailability of essential minerals and binds micronutrients (e.g., Fe, Zn, K, Cu, Co, Mg, and Ca). Furthermore, food allergies to plant protein need to be addressed, since plant proteins, especially legume proteins themselves contain some allergens.

Interestingly, when compared with natural beef, plant-based meat analogues have more energy value, total fats, saturated fats, and Na and Fe contents [14], perhaps because of the addition of excess fat and/or oil (e.g., coconut oil and cocoa butter) for mimicking animal fat, coloring agents, and spices to the meat analogues during the processing of plant proteins (Table 3). These results reveal that manufacture of plant-based meat analogues may reduce the benefits of nutrients present in the original plant protein itself. In the absence of such a processing step, fat and saturated fat contents of plant protein varied from 0.5 to 8 and 0 to 0.9 g/100 g, respectively [32]. Nevertheless, the challenges can be overcome by advanced technological development, and plant-based meat analogues will be important protein sources in the future.

### Edible insects

#### Definition and present features

Insects are one of the largest living resources on the earth, with a total of 5.5 million species [33]. Among them, almost 2,000 species of insects are consumed in 113 countries, especially Africa, South America, and Southeast Asia [34,35]. In such regions, eating insects is an ancient custom (so-called entomophagy) from at least 3,000 years ago. Insects have been used as a valuable protein resource for their high protein content with essential amino acids sufficient for our daily requirement [36–38]. The most frequently consumed species of insects are *coleoptera* (beetles), *lepidoptera* (caterpillars), *hymenoptera* (ants, wasps, and bees), *orthoptera* (locusts, grasshoppers, and crickets), *hemiptera* (leafhoppers, planthoppers, and cicadas), *isoptera* (termites), *odonata* (dragonflies), and *diptera* (flies) [39,40].

However, the acceptance of eating insects is low in western consumers, mostly because of a negative image regarding insects, especially as a food component. Therefore, entomophagy has decreased in our diet, as various food product options are increasingly available with the development of food science and technology [36,37,41]. Consequently, there is an urgent need for meat alternatives due to the importance of traditional meat as a main diet in our lives [42]. Nonetheless, the importance of edible insects has emerged because of the increasing need for meat alternatives for proteins. In recent years, the market for insects is steadily increasing and is expected to exceed $ 522 million US dollars by 2023 [43].

### Benefits as meat alternatives

The major purpose of the consumption of insects by humans is to provide an excellent source of proteins. The nutritional values of edible insects vary depending on their species, sex, metamorphosis state (e.g., larvae, pupae, and adults), origin, diet, and different methods of processing due to their large diversities (Table 4) [35,44]. Xiaoming et al [45] reported that protein content in 100 different species ranged from 13% to 77% on the basis of their dry matter. Analyses of 87 insect species in Mexico revealed a protein content of 15% to 81% with high digestibility [46]. de Castro et al [44] reviewed the nutritional value of frequently consumed insects (e.g., beetles, files, bugs, bees, wasps, sawflies and ants, butterflies and moths, grasshoppers, crickets, and locusts) and found large variations (1% to 81%) among the protein contents. The bioavailability of insect protein is also high with good digestibility (76% to 96%), which is a little less than that of egg or beef protein (95% and 98%, respectively) [35,47]. Thus, undoubtedly, insects can serve as a fine protein source in our diet. In Central Africa, there was a time when about 50% of dietary proteins were obtained from insects [42]. Compared to plant protein, insect protein has nutritional benefits with respect to total protein levels, essential amino acids, and bioavailability. Kouřimská and Adámková [35] stated that some species of insects have high lysine, tryptophan, and threonine contents, which are not found in some plants.

Edible insects can provide other beneficial nutrients such as fats with highly unsaturated fatty acids, vitamins, and minerals [35,44]. In insects, fat is the second abundant nutrient (approximately 10% to 60%, on the basis of dry matter) followed by proteins. In general, the fats can be classified into 80% triglycerides and 20% phospholipids, which play a role in energy reserves, cell membrane structure, and regulatory physiology [35,48]. The profile of unsaturated fatty acids in edible insects is comparable to that of poultry and fish; however, insects have more polyunsaturated fatty acids [42,49]. Rumpold and Schlüter [49] reported that major omega-3 fatty acids, including eicosapentaenoic acid and docosahexaenoic acid, were not detected in most insects; however, their levels could be increased with feed modifications during insect rearing. In addition, edible insects are rich in Fe, Zn, Na, Ca, P, Mg, Mn, Cu, riboflavin, pantothenic acid, and biotin [49,50].

The benefits of edible insects are not only limited to their high nutritional content, but also to high feed/meat conversion rate and lower requirements of land, water, and feed [44,51,52]. In addition, they have a high fecundity rate with year-round breeding and small space requirements. In some species (e.g., palm weevil larvae), the byproducts can be used for other livestock and/or humans, resulting in high recycling capability.

### Research trends and challenges

Many studies have been conducted on the use of edible insects as human food or ingredients. However, despite constant efforts to expand their market and consumption, eating insects may not become a mainstream dining option [43]. People are hesitant to consume insects owing to a skeptical attitude towards novel foods [42,52]; This is a part of food neophobia, which can determine the acceptance of edible insects as meat alternatives [53]. Consumers who have not experienced consuming edible insects perceive insects to be dirty, disgusting, and dangerous, ultimately rejecting them as a food resource [54]. This phenomenon is a main challenge for consumption of edible insects, especially in Western countries [43]. According to Verbeke [55], only a few consumers (12.8% males and 6.3% females) in the Western society accept edible insects as a food item. Post [56] also reported that most of the insects in the Netherlands are used as a pet food rather than human diet. To overcome food neophobia related to insects, regular inclusion of insects in the daily diet can be helpful, while increasing its positive perception [44,52]. Imparting information on the benefits of edible insects on the aspects of nutrition, environment, and culture is considered another solution [51,57]. However, its actual effect is still negligible, and the Western civilization is not ready to eat edible insects in intact forms [52].

The development of insect-based ingredients/products rather than intact forms can facilitate the adoption of insects as a food resource [57–59]. Therefore, several studies have been conducted to process insects as new food ingredients and to include them in familiar foods or in processing of food products [36,37]. These methods involve raw material processing, protein processing, and oil processing [53], which can improve the quality characteristics (e.g., flavor) and functional properties (e.g., angiotensin I converting enzyme inhibitory activity and antimicrobial and antioxidant functions) when applied to food ingredients [44,60]. Recently, raw material processing through drying and/or milling is the most widely used method for applying edible insects as a food product. When insects are converted to a dry powder, their volume is lower than that of the original product, resulting in easier transportation; in addition, the product can be stored for a long time owing to low water activity. Meanwhile, protein and oil-processing methods have been investigated to extract proteins and oils from insects. These extracting processing not only enhance nutritional values but also increase technical functional properties [37]. Various edible insects are added as ingredients to foods such as bread, cookies, and sausages to enhance their nutritional value and food quality. Therefore, insects can be used without their negative image impact to enhance the aforementioned properties of food products [52].

Nonetheless, safety issues of edible insects, such as anti-nutrients (e.g., chitin and toxic substances [cryptotoxics and phanerotoxics]), microbial risk, and allergens, still exist [42, 44]. Sufficient data to confirm the safety of anti-nutrients in insects should be obtained in future studies. In particular, since studies on food allergies of insects are limited, further investigations are needed for the growth of the edible insect industry [39]. Till date, some allergic cross-reactive proteins of arthropods (arachnids and crustaceans) are known [61].

### Cultured meat

#### Definition and present features

Cultured meat (also called *in vitro* meat, synthetic meat, lab-grown meat, bioartificial muscle, and Frankenstein meat) is the latest emerging meat alternative. It can be defined as artificial meat produced using stem cell technology [62]. The idea of cultured meat was first mentioned in 1932 by Winston Churchill, a previous prime minister of UK. Cell and tissue engineering techniques have been developed for medical purposes. However, recently, because of advanced technological inputs, they have been applied in the field of food technology [63,64] for large-scale culturing [56]. Based on such developments, the first beef patty cultured from bovine muscle cell was introduced to the public in 2013. The patty was made of muscle cells with the addition of beet juice and saffron to make a meat-like product; however, production cost was extremely high [63, 65].

So far, cultured meat could not be commercialized owing to technical difficulties in its mass production and cost. The patty (approximately 85 g) made by Dr. Post required US $330,000 in 2013, and a meatball (approximately 1 kg), which was recently unveiled by Memphis Meat, cost US $40,000 [63, 65]. Therefore, to launch cultured meat in the market, its production cost should be lowered, and quality characteristics should be improved. The optimization process should be preceded by the whole process of cultured meat [66]. Once cultured meat is produced with a similar quality to that of traditional meat, it may play an important role in increasing meat supplies because it will be the only actual meat that has animal protein [67,68]. Mosameat, Memphis Meats, Super Meat, Integriculture, Just, and others are major companies manufacturing cultured meat; they are planning to release their products from 2021. Various types of cultured meat, such as meatballs, burgers, and sausages may be launched, and their market size is expected to be US $4.3 million for meatballs, US $3.7 million for burgers, and US $3.3 million for sausages. One of the articles reported that the appearance of cultured meat is expected to change the trends in global meat market, as it is expected to occupy 35% of the global meat market in the next 20 years [69].

### Benefits as meat alternatives

The biggest merits of cultured meat are its similarities to traditional meat, as it is derived from farm animals, and may be environmentally sustainable [67,68]. This product can meet both the nutritional and sensory preferences of consumers because of its superior taste and texture than other meat alternatives [62]. In this respect, cultured meat can attract consumers who do not want to change their traditional diet style of meat consumption. Besides, according to Zhang et al [68], during the production of cultured meat, a single cell can proliferate several times; therefore, fewer numbers of animals are needed than in livestock farming.

In addition, there are other advantages of cultured meat. Bhat et al [63] suggested that cultured meat may be utilized for several other applications, such as creation of functional and designer meat, quick production, availability of exotic meat, vegan meat, efficient nutrient, and energy conversion. Besides, the benefits of cultured meat include public support, animal welfare, reduction in zoonotic and food borne disease, reduction in resource use and ecological foot print, and reforestation and wild life protection; it can also be used for space missions and settlements. Although the development of cultured meat is still in progress, it may be possible to control the ingredients in the products to have more health benefits without long farming processes [63]. In addition, all processes in culturing meat are conducted under sterile conditions employing various food quality and safety management systems such as Good Manufacturing Practice and Hazard Analysis and Critical Control Points. Therefore, it is possible to produce safer products devoid of hazards such as contamination, antibiotic abuse, infectious diseases, and food poisoning [68].

### Research trends and challenges

Although cultured meat is about to be released in a few years, technologies for its processing are still insufficient. The most urgent challenge could possibly be the development and optimization of mass production process with reasonable pricing. From the choice of cells to tissue engineering techniques (Table 5) (See Specht et al [70] for more detailed information), uncertainties in cell culture and muscle development should be studied and further optimized for the mass production of cultured meat [63,65]. Gaydhane et al [71] suggested cells, culture media, scaffolds, bioreactors, culture conditions, and processing (also called mimicking) as the key factors for producing cultured meat; this report mostly agrees with other studies [68,72–74]. As the range of studies conducted on such factors is quite wide and comprehensive details are not yet clear, only a brief introduction on the culture media, scaffolds, and bioreactors will be discussed in this review based on the currently-available literatures.

During cell culture, optimal formulation of culture media is important, as it can affect growth rate of cells [71]. Culture media contain various nutrients, hormones, sera with growth factors, and other components for cell growth [73]. Among them, the use of serum (e.g., fetal bovine serum, horse serum) in culture media is a cause for concern. Serum is a necessary component in culture media, as it can facilitate the growth of muscle satellite cells. However, researchers have suggested that its use in culture media should be replaced or eliminated, as it is variable and expensive and is a main reason of high production cost of cultured meat [67]. In addition, its production process may not be ethical and sustainable, as it is derived from calves. Therefore, alternative ingredients for serum in culture media, especially serum-free media, have been one of the main research areas for cultured meat.

Bioreactor and scaffolds are other important factors in mass production of cultured meat [75]. In general, a bioreactor is applied for large-scale cell growth under controlled conditions of temperature, pH, oxygen partial pressure, and shear stress, providing a more homogeneous environment during cell proliferation and/or differentiation with detailed monitoring of its conditions [76,77]. Previous studies have reported that different types and conditions of bioreactors can affect mass production of cultured meat. In the last few years, various types of bioreactors (e.g., stirred tank bioreactor [a.k.a. spinner flask], High-Aspect-Ratio-Vessel bioreactor, fluidized bed bioreactor, hollow fiber bioreactors, and packed bed bioreactors) have been developed with different sizes [76,78]. Moreover, not only temperature and pH, but also oxygen partial pressure and shear stress are important for optimal conditions of a bioreactor. For example, low oxygen partial pressure decreases the differentiation rate of cells, but increases their proliferation. In the case of shear stress, its application with increasing impeller size and rpm as well as its location and the internal vessel used can affect cell damage. Therefore, low shear stress and stable oxygen perfusion should be set up in a bioreactor even at large volumes [72]. Furthermore, efficiencies of bioreactors varied for different cell lines. Therefore, customized bioreactors and their proper use should be investigated for optimization of mass production of cultured meat.

Scaffolding is a method that can impart more meat-like texture to cultured meat instead of complex co-culture of connective tissue [79]. Scaffolds consist of biopolymers, and their application is known to be best suited for cultured meat. Cell-attached scaffolds are suspended in a bioreactor with culture media, producing cultured meat on a large-scale [64,68]. When considering the requirements for scaffolds, collagen is the most frequently used material, and plant-based sources (e.g., alginate, cellulose, or chitosan) have also been developed [79]. However, so far, scaffolding cannot be used to prepare a highly-structured product and is only capable of producing ground and/or emulsified products; therefore, improvement of a highly-developed structure of cultured meat is one of the challenges in future [63,72].

## CONCLUSION

**Figure 2:**
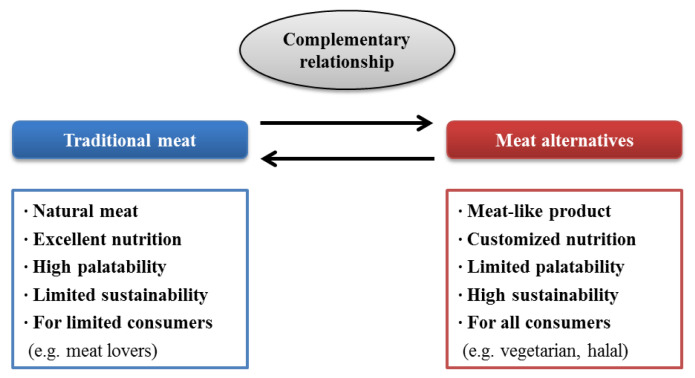
Final goal of traditional meat and meat alternatives in the future.

There is no doubt that livestock-based traditional meat and meat products are the best protein sources, with excellent palatability and ample consumption. However, changes in consumers’ perception and the value of land/water resources and environmental sustainability will lead to the development of meat alternatives. Consequently, to conserve the limited supply of traditional meat, meat alternatives, including plant-based meat analogues, edible insects, and cultured meat, will play important roles, depending on the degree of their technical development and consumer acceptance, while maintaining a complementary relationship with traditional meat (Figure 2).

## Figures and Tables

**Table 1:** Types and definition of meat alternatives as a protein source

Types	Definition
Conventional meat	Traditional meat from farm animals
Plant-based meat analogue	Meat analogue made of plant and fungus proteins
Edible insect	Insect used as food resources
Cultured meat	Artificial meat produced using stem cell technology
Modified meat	Meat from genetically modified animals
3D-printed meat	Fabricated meat made of native or non-native food materials with 3D printing system

Modified from Bonny et al [10]; Dick et al [80].

**Table 2:** Plant proteins used for plant-based meat analogues

Plant	Protein
Wheat, rye, and barley	Gluten (Gliadins, Glutenins)
Soybean	β-conglycinin
Legumes	Glycinin, Vicilin
Oil seeds	Legumin, Albumins, Globulins, Glutelins
*Fusarium venenatum* (Filamentous fungus)	Mycoprotein

Adapted from Asgar et al [13].

**Table 3:** Ingredient used during the manufacture of plant-based meat analogues

Ingredient	Purpose	Usage level (%)
Water	Ingredient distribution Emulsification, juiciness, cost	50–80
Textured vegetable proteins	Water binding, Texture/mouthfeel Appearance; protein fortification/nutrition Source of insoluble fiber	10–25
Non-textured proteins	Water binding, emulsification Texture/mouthfeel Protein fortification/nutrition	4–20
Flavors/spices	Flavor: savory, meaty, roasted, fatty, serumy Flavor enhancement (for example, salt) Mask cereal notes	3–10
Fat/oil	Flavor, texture/mouthfeel Succulence, Maillard reaction/browning	0–15
Binding agents	Texture/“bite,” water binding, may contribute to fiber content, can determine production processing conditions	1–5
Coloring agents	Appearance/eye appeal Natural or artificial	0–0.5

Adapted from Asgar et al [13].

**Table 4:** Nutrient composition of edible insects depending on different species (on a dry matter basis)

Edible insects	Protein (%)	Fat (%)	Fiber (%)	NFE (%)	Ash (%)	Energy content (Kcal/100 g)
Blattodea (cockroaches)	57.30	29.90	5.31	4.53	2.94	-
Coleoptera (beetles, grubs)	40.69	33.40	10.74	13.20	5.07	490.30
Diptera (flies)	49.48	22.75	13.56	6.01	10.31	409.78
Hemiptera (true bugs)	48.33	30.26	12.40	6.08	5.03	478.99
Hymenoptera (ants, bees)	46.47	25.09	5.71	20.25	3.51	484.45
Isoptera (termites)	35.34	32.74	5.06	22.84	5.88	-
Lepidoptera (butterflies, moths)	45.38	27.66	6.60	18.76	4.51	508.89
Odonata (dragonflies, damselflies)	55.23	19.83	11.79	4.63	8.53	431.33
Orthoptera (crickets, grasshoppers, locusts)	61.32	13.41	9.55	12.98	3.85	426.25

Modified from Rumpold and Schlüter [49].

**Table 5:** Critical technology elements of cultured meat

Critical technology elements	Design requirements for cultured meat	Relevant technologies and advances within the cell-based therapeutics industry
Cell line	Derived from agriculturally-relevant species Capable of differentiation into meat-relevant cell types (muscle, fat, fibroblast, etc.) Genetically stable and immortalized Optimized for large-scale growth (tolerate suspension, controlled differentiation, etc.)	Development of small molecule cocktails that can replace the need for genetic approaches to induce pluripotency and to facilitate maintenance of pluripotency Footprint-free methods of cell line engineering using RNA or protein delivery or excisable transposons Improved protocols for cell freezing to maintain viability and phenotypic fidelity
Culture media	Animal component-free, antibiotic-free, ideally chemically defined Optimized for meat-relevant cell lines and co-culture of multiple cell types Extremely low cost and high-volume production capacity Engineered or synthetic growth factors	Development of methods for streamlining iterative optimization of animal component-free media formulations Immobilizing growth factors on beads to prevent depletion in the media via perfusion
Scaffolding	Edible and/or biodegradable and food grade materials Support cell adherence Support vascularization and media perfusion Biomechanical properties suitable for tissue maturation Scalable production capacity	Biocompatible, non-animal-derived scaffolding materials pioneered in the regenerative medicine field Use of tunable scaffold parameters (stiffness, etc.) to spatially direct differentiation Degradable materials that enable cell migration and vascularization after patient implantation
Bioreactors	Support cell proliferation as well as tissue maturation/perfusion Large volume, low maintenance High-yield cell harvesting Real-time, in-line cell monitoring for quality control Integrated media filtration and recycling system Highly automated; closed system	Integrated, closed systems with increasing automation to reduce errors and contamination risk associated with human handling In-line monitoring of media components to adjust perfusion in real time Novel technologies to improve efficiency of cell separation and harvesting

Adapted from Specht et al [70].
